# Vesicular transport of a ribonucleoprotein to mitochondria

**DOI:** 10.1242/bio.20149076

**Published:** 2014-10-17

**Authors:** Joyita Mukherjee, Biraj Mahato, Samit Adhya

**Affiliations:** Genetic Engineering Laboratory, CSIR–Indian Institute of Chemical Biology, 4 Raja S. C. Mullick Road, Calcutta 700032, India; * Present address: Penn Institute for Regenerative Medicine, University of Pennsylvania, 3800 Spruce Street, Philadelphia, PA 19104, USA.

**Keywords:** RNA protein complex, Endosome, Sorting, Caveolin 1, Mitochondria

## Abstract

Intracellular trafficking of viruses and proteins commonly occurs via the early endosome in a process involving Rab5. The RNA Import Complex (RIC)-RNA complex is taken up by mammalian cells and targeted to mitochondria. Through RNA interference, it was shown that mito-targeting of the ribonucleoprotein (RNP) was dependent on caveolin 1 (Cav1), dynamin 2, Filamin A and NSF. Although a minor fraction of the RNP was transported to endosomes in a Rab5-dependent manner, mito-targeting was independent of Rab5 or other endosomal proteins, suggesting that endosomal uptake and mito-targeting occur independently. Sequential immunoprecipitation of the cytosolic vesicles showed the sorting of the RNP away from Cav1 in a process that was independent of the endosomal effector EEA1 but sensitive to nocodazole. However, the RNP was in two types of vesicle with or without Cav1, with membrane-bound, asymmetrically orientated RIC and entrapped RNA, but no endosomal components, suggesting vesicular sorting rather than escape of free RNP from endosomes. In vitro, RNP was directly transferred from the Type 2 vesicles to mitochondria. Live-cell imaging captured spherical Cav1^−^ RNP vesicles emerging from the fission of large Cav^+^ particles. Thus, RNP appears to traffic by a different route than the classical Rab5-dependent pathway of viral transport.

## INTRODUCTION

Many viruses, proteins and lipids are taken up through, or recycled from, the plasma membrane through endocytosis. A number of distinct uptake mechanisms have been described, including through clathrin-coated pits, caveole/lipid rafts and clathrin- or caveolin-independent pathways (Wieffer et al., 2009; Hansen and Nichols, 2009). Endocytic vesicles from the plasma membrane enter pre-existing sorting compartments from which the cargo emerges either free or in secondary vesicles that are targeted to the destination compartment.

The transport of various cellular RNAs within and between the nuclear and cytosolic compartments involves ribonucleoprotein (RNP) complexes, motor proteins and cytoskeletal elements (Eliscovich et al., 2013). Recently, the transport of RNA entrapped in membrane vesicles (exosomes and microvesicles) has been described (Raposo and Stoorvogel, 2013). Exosomes originate within endosomes and are trafficked to the plasma membrane by a poorly understood pathway (Raposo and Stoorvogel, 2013). DNA-cationic liposome complexes are trafficked by endocytic pathways to the nucleus (Wong et al., 2007). We have previously shown that a RNP derived from the protozoal parasite *Leishmania tropica* is taken up by mammalian cells and targeted to mitochondria (Mahata et al., 2006; Mukherjee et al., 2007; Mahato et al., 2011). The protein component of this RNP is the RNA Import Complex (RIC), a multisubunit complex that induces RNA import into mitochondria (Adhya, 2008). However, the trafficking route of RIC is unknown. Here we have employed genetic, cell biological and biochemical approaches to study the steps of the transport pathway of RIC in cultured cells and in cell free systems.

## RESULTS

### Trafficking of RNA to mitochondria

**Fig. 1.**
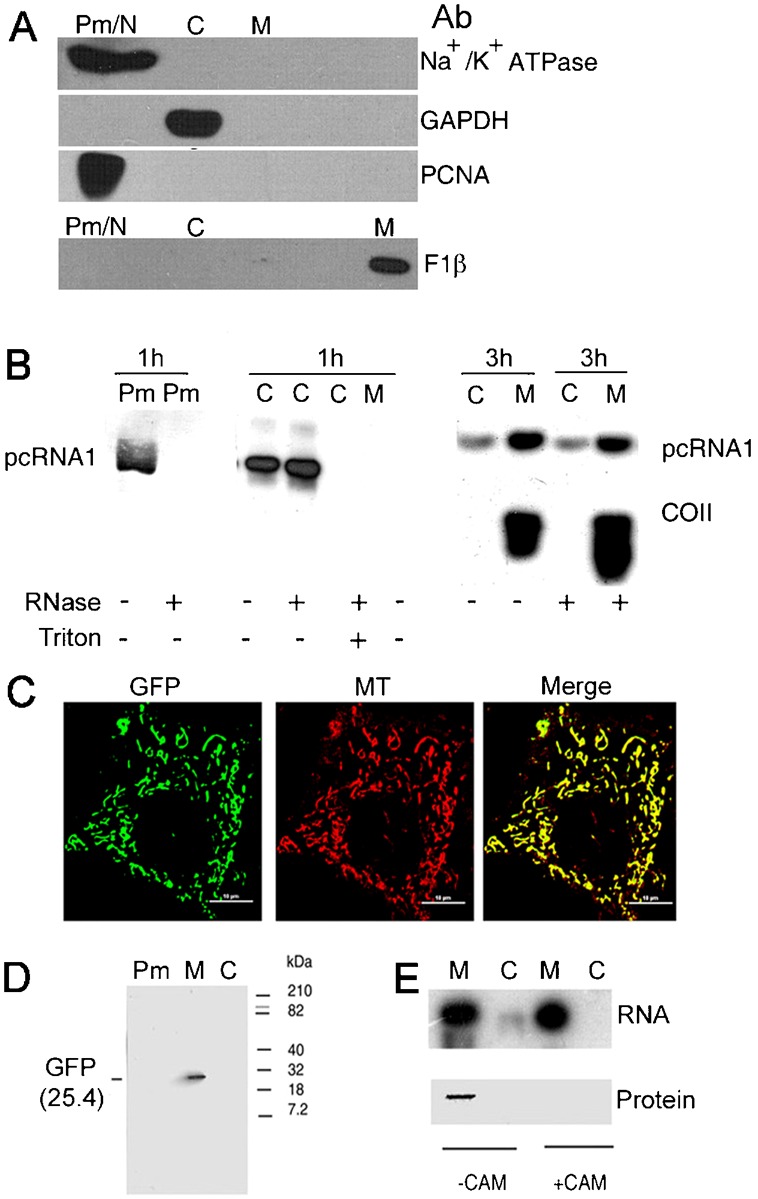
Trafficking of RNAs to mitochondria. (A) Western blot of Plasma membrane/Nuclear (Pm/N), cytosolic (C) and mitochondrial (M) subcellular fractions from HepG2 cells probed with antibodies against marker proteins: Na–K ATPase (Plasma membrane), PCNA (nucleus), GAPDH (cytosol) and F1 ATPase β subunit (mitochondria). Lack of cross-contamination in the cytosolic and mitochondrial fractions is evident. (B) FLP32.39 cells were incubated with pcRNA1-R8 complex for the indicated times according to the trafficking time course protocol (Materials and Methods), separated into subcellular fractions, the indicated fraction was incubated in the absence or presence of RNase, without or with Triton X-100 as shown, and the RNA analyzed by Northern blot using anti-COII probe. (C–E) Expression of tagged GFP RNA in mitochondria. (C) Live FLP32.39 cell transfected with GFP RNA for 24 h and stained with MitoTracker Deep Red (red), expressing GFP fluorescence (green) in mitochondria. (D) Western blot of indicated subcellular fractions of GFP RNA-transfected FLP32.39 cells probed with anti-GFP antibody. (E) Northern (upper) and Western (lower) blots of indicated fractions of GFP RNA-transfected FLP32.39 cells incubated in the absence (−CAM) or presence (+CAM) of chloramphenicol (100 µg/ml). Scale bar: 10 µm.

Cultured cells were incubated with the pcRNA1-RIC complex. At various times, subcellular fractions (plasma membrane, mitochondrial and cytosolic) were isolated and probed for the presence of RNA or of subunits of the carrier complex. The purity of the fractions was assessed by Western blot using marker antibodies (Fig. 1A). The low-speed pellet (Pm/N) was positive for both plasma membrane (Na–K ATPase) and nuclear (PCNA) markers, but negative for cytosolic (GAPDH) and mitochondrial (F1 ATP synthase β subunit); the high-speed pellet (M) was specific for mitochondrial proteins, while the high speed supernatant (C) was positive only for cytosolic markers. This shows the lack of significant cross-contamination of the subcellular fractions obtained by the differential centrifugation method described (Materials and Methods).

After 1 h of incubation with RNP, the 4.1-kb RNA was localized in the plasma membrane and cytosolic fractions, but not in mitochondria of cybrid FLP32.39 containing a 1.9-kb mtDNA deletion (Fig. 1B); in the mitochondrial fraction, mature COII mRNA was absent due to the deletion of the gene (Mahato et al., 2011). After 3 h, the amount of RNA in the cytosolic fraction was much reduced, while the RNA was predominantly in the mitochondrial fraction. Within mitochondria, the polycistronic precursor was found, as well as mature COII mRNA (Fig. 1B), arising out of the processing of the polycistronic precursor, as described previously (Mahato et al., 2011). To determine whether the RNA was exposed to the environment during transit, each fraction was treated with ribonuclease (RNase). While the plasma membrane associated RNA was completely nuclease sensitive, RNA in the post-mitochondrial fraction was almost completely resistant (Fig. 1B). In presence of detergent Triton X-100, this RNA became RNase-sensitive (Fig. 1B). This result is consistent with the exposure of pcRNA on the surface of plasma membrane derived vesicles, and within cytoplasmic membrane-bound compartments, prior to its translocation to mitochondria. In the mitochondrial fraction at 3 h, the pcRNA and mature mRNAs were also RNase-resistant, as expected (Fig. 1B). These observations indicate the presence of an intermediate cytosolic compartment or vesicle with entrapped RNA during transport to mitochondria.

We additionally tested for the transport of tagged Green Fluorescent Protein (GFP) reporter RNA by direct microscopic examination. In live cells treated with tagged GFP RNA, intense mitochondrial GFP fluorescence was observed, as indicated by co-localization of GFP with MitoTracker Deep Red (Fig. 1C). Full-length GFP was present in Western blots of mitochondrial protein (Fig. 1D). Formation of GFP was sensitive to chloramphenicol, indicating mitochondrial translation of GFP RNA (Fig. 1E).

### Dependence of mitochondrial targeting of RIC-RNP on endocytic factors

To assess the factors involved in trafficking of RIC-RNP to mitochondria, cells were treated with siRNAs targeted against individual endocytic factors and transport of RIC-RNP assayed in three different ways: (1) the expression of Green Fluorescent protein (GFP) in mitochondria of cells transfected with GFP RNA; (2) rescue of respiration in cells with a 4.1-kb mtDNA deletion (and hence respiration-deficient) by a complementary polycistronic transcript (pcRNA1; Mahato et al., 2011); and (3) co-localization of fluorescent pcRNA1 with MitoTracker Deep Red in live cells by confocal microscopy.

**Fig. 2.**
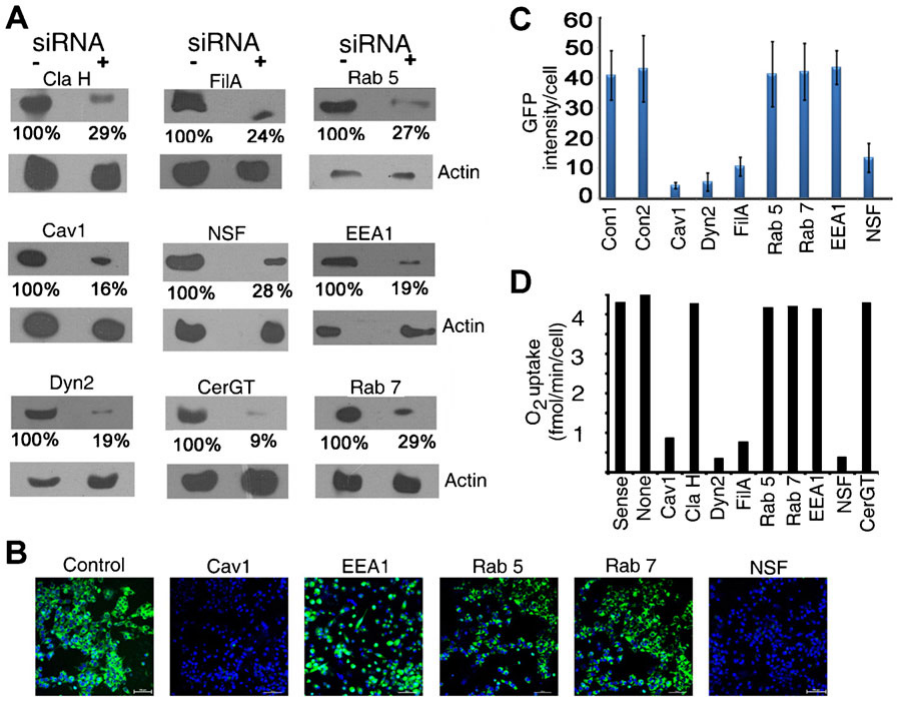
Effect of siRNA-mediated knockdown (KD) of endocytic factors on RNP trafficking. HepG2 (A–C) or FLP32.39 (D) cells were transfected with control RNA (Cav1 sense) or gene-targeted siRNA as indicated for 48–72 h, then treated with GFP RNA-RIC complex (A–C) for 24 h or pcRNA1-RIC (D) for 6 h. (A) Western blots showing the efficiency of KD. (B) GFP fluorescence in live cells. (C) Quantification of GFP intensity (arbitrary units)/cell in control and KD cells. (D) O_2_ uptake by control or siRNA-treated FLP32.39 cells in presence of pcRNA1. Scale bar: 10 µm.

In cells transfected with siRNAs, the targeted protein was down regulated by 70–90% after 24 h of siRNA treatment (Fig. 2A). Non-targeted proteins such as actin were not affected. Caveolin 1 (Cav1) sense or scrambled siRNA had no effect (data not shown). The GFP fluorescence per cell was reduced by depletion of Cav1, Dynamin 2 (Dyn2), Filamin A (FilA) and NSF, but not by knockdown of EEA1, Rab5 or Rab7 (Fig. 2B,C). Respiration rescue of FLP32.39 was identically affected by siRNA against these proteins, but depletion of Clathrin Heavy Chain had no effect (Fig. 2D).

Import and mitochondrial targeting in siRNA-treated cells were also assessed by confocal microscopy using fluorescent pcRNA1 (supplementary material Fig. S1). Uptake and co-localization indices were identically affected by knockdown (KD) of Cav1, filamin A and dynamin2, but not by KD of Clathrin, EEA1, Rab5, or Rab7 (supplementary material Fig. S1). In case of NSF-KD, uptake was normal but the RNA did not localize to mitochondria (supplementary material Fig. S1), indicating a block at an intermediate step.

Taken together, these results indicate that endocytosis of the RNP into cytosolic transport vesicles is dependent on Cav1, Dyn2 and FilA, but independent of clathrin. Dyn2 is required for formation of caveolin as well as clathrin coated vesicles (Henley et al., 1998), while FilA is involved in the transport of caveolar endocytic vesicles on actin microfilaments (Stahlhut and van Deurs, 2000). Caveolin is associated with sterol- and glycolipid-enriched lipid raft domains on the plasma membrane (Parton and Richards, 2003). GM1, a raft glycolipid, acts as the receptor for various caveolar cargoes such as SV40 virus; siRNA-mediated down-regulation of ceramide glucosyl transferase, an enzyme required for biosynthesis of the oligosaccharide moiety of GM1 and other membrane glycolipids (Sandhoff and Kolter, 2003), results in inhibition of internalization of SV40 by the caveolar pathway (Pelkmans et al., 2005). However, down-regulation of this enzyme had no effect on the internalization or targeting of RNP (Fig. 2D; supplementary material Fig. S1).

The most widely studied trafficking pathway for cargo destined for the ER or Golgi apparatus, or for recycling to the plasma membrane, involves fusion of endocytic vesicles to the Early Endosome (EE), a pre-existing organelle that matures to form Late Endosomes (LE). Sorting of cargo can occur at either EE or LE. Trafficking through EE is dependent on the Rab5 GTPase and its effector EEA1, both of which are located on the EE membrane, while formation and transport of LE and cargo sorting within this organelle are dependent on Rab7 (Huotari and Helenius, 2011). Transport of RIC-RNP to mitochondria was not affected by depletion of either Rab5 or EEA1 (Fig. 2A–C; supplementary material Fig. S1). Transport to mitochondria was also not affected by KD of Rab7, a LE component required for sorting (Fig. 2B–D; supplementary material Fig. S1).

### RNP vesicles in the cytoplasm

**Fig. 3.**
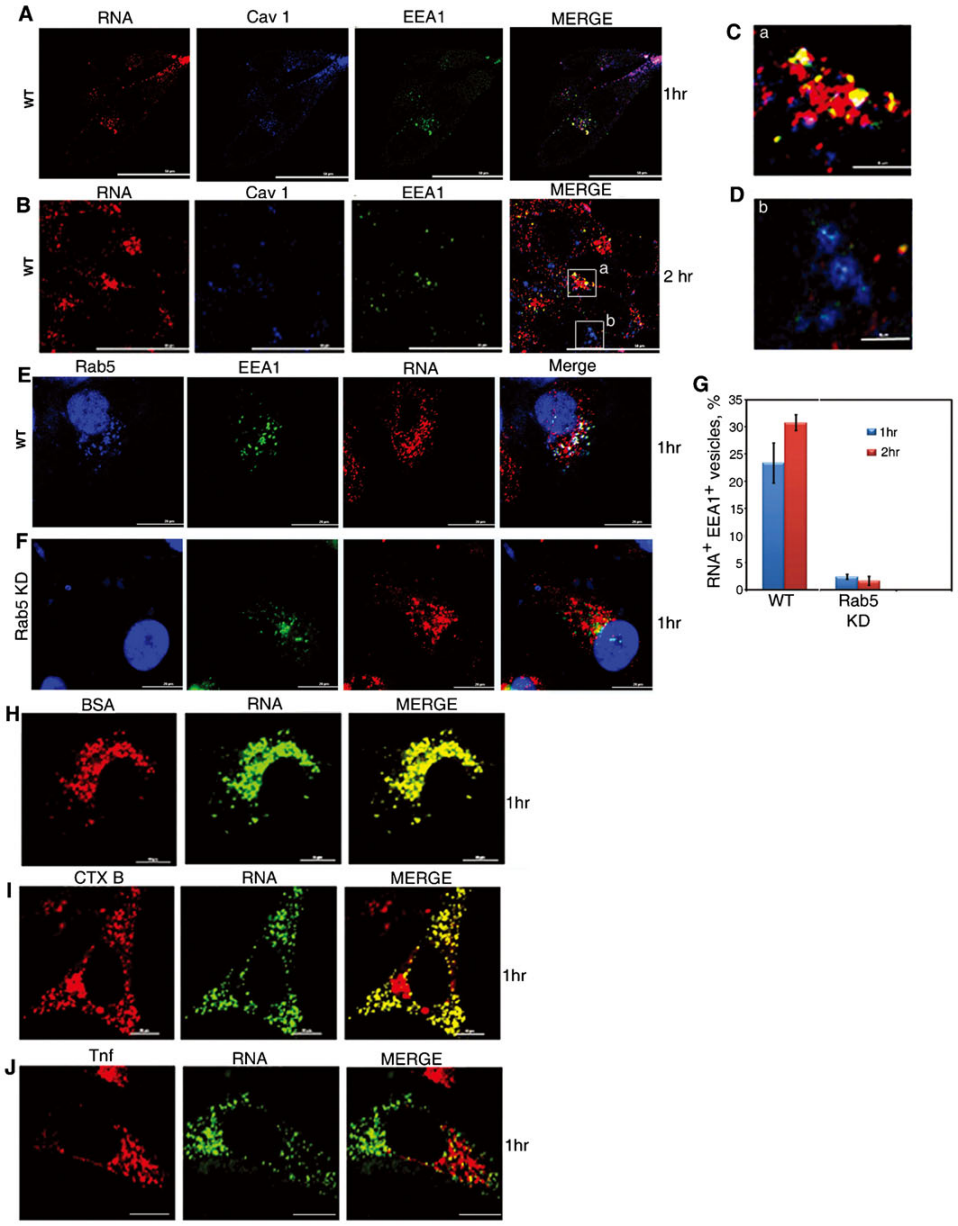
RNP vesicles in cells. (A–G) HepG2 cells were transfected with AF546-labeled pcRNA1 (red) for indicated times, fixed and stained for Cav1 (blue), EEA1 (green), or Rab5 (blue). In E, F, nuclei were DAPI-stained. (C,D) show enlarged areas a and b, respectively, boxed in the merged image of panel B. (E, F) Co-localization of RNA with EEA1-positive endosomes in normal (E) or Rab5-KD(F) cells. (G) Per cent of EEA1-positive RNP vesicles in normal or Rab5-KD cells after 1 h (blue bars) or 2 h (red bars) of treatment with pcRNA1. (H–J) Co-transport of RNP and known endocytic cargo. HepG2 cells were incubated with a mixture of AF488-RNP (1 pmol) and AF647-labeled, albumin (H), cholera toxin B subunit (I) or transferrin (J) for the indicated times and imaged live. Scale bars: 50 µm (A,B); 6 µm (C,D); 20 µm (E,F); 10 µm (H–J).

In RNP-transfected cells, numerous RNA-containing vesicles of heterogeneous size were observed in the cytoplasm at different times (Fig. 3). To study the nature and distribution of the RNP vesicles in the cytosol, and their relationship with endosomes, cells transfected with Alexa Fluor 546 labeled pcRNA1 for various times were stained for Cav1 and/or the early endosome marker EEA1. After 1 h of treatment, the RNP was localized in patches on the plasma membrane, with evidence of large aggregates (capping) in some cells, and in small intracellular Cav1^+^ vesicles (Fig. 3A,B). Additionally, Cav1^+^RNA^+^EEA1^+^ vesicles were observed (accounting for 25–30% of the total RNA), indicating fusion of some of the transport vesicles with the early endosome (Fig. 3B). Co-localization of RNA to endosomes was reduced to insignificance in Rab5-KD cells (Fig. 3E–G). Thus a fraction of the RNP is trafficked to endosomes in a Rab5-dependent manner, whereas trafficking to mitochondria is Rab5-independent (Fig. 2).

At 2 h post-transfection, different RNP vesicles were evident upon examination of a large number of cells stained for Cav1 and/or EEA1: RNA^+^Cav1^+^ vesicles scattered throughout the cytoplasm or concentrated near the nucleus; perinuclear Cav1^−^ clusters of large vesicles (Fig. 3B,C); and small Cav1^−^ vesicles scattered throughout the cytoplasm. These vesicles were negative for EEA1 (Fig. 3B) or LAMP1 (supplementary material Fig. S2). Large EEA1^+^ endosomes with intraluminal Cav1^+^ vesicles (ILV), but lacking RNA, were observed in the latter cells (Fig. 3D), indicating the formation of maturing endosomes recycling Cav1 and degrading the RNP.

In live cells treated with a mixture of RNP and either albumin or the B subunit of cholera toxin (CTX-B), both of which are known to be endocytosed via caveolae (Parton and Richards, 2003; Minshall et al., 2000), large RNP vesicles containing albumin (Fig. 3H) or CTX-B (Fig. 3I) were observed. In the latter cells, a significant amount of CTX-B was observed in a cluster of perinuclear vesicles resembling endosomes, a normal sorting compartment for the toxin. The RNP vesicles, however, were quite distinct from endosomes loaded with transferrin (Fig. 3J).

### Immunoselection of cytosolic RNP

**Fig. 4.**
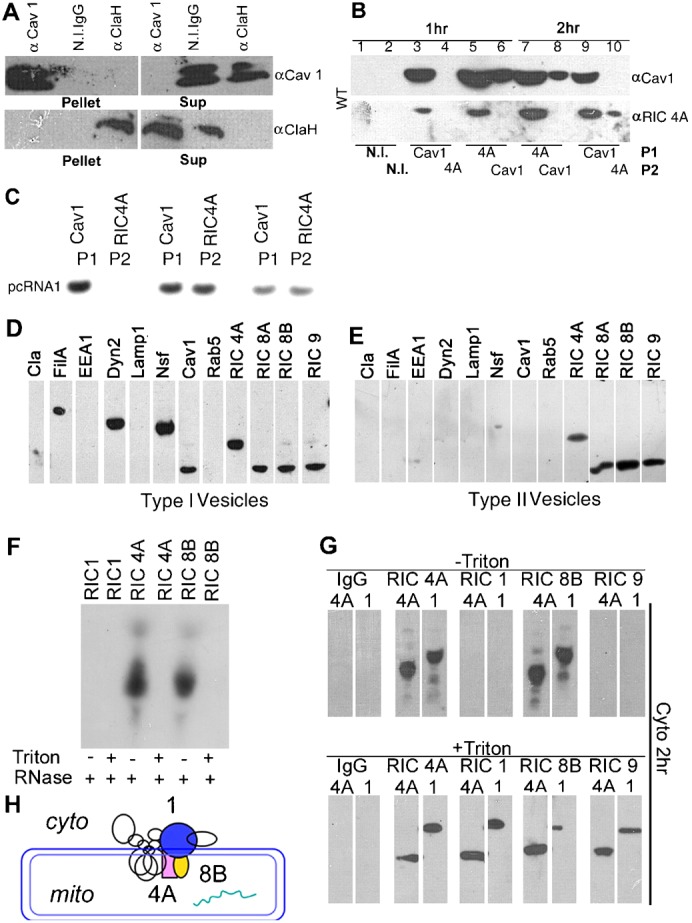
Intermediate vesicles isolated by immune-selection. (A) The cytosolic fraction (40 µg protein) from HepG2 cells transfected with pcRNA1 for 1 h was subjected to immunoprecipitation (IP) with the indicated antibody or non-immune (N.I.) IgG and the pellet or supernatant (sup) was analyzed by Western blot using anti-Cav1 (upper) or anti-ClaH antibody (lower). (B,C), sequential immunoprecipitates (P1 and P2) obtained with the indicated antibodies from cytosolic fractions of cells treated for the indicated times with pcRNA1-RIC complex were analyzed by Western (B) or Northern (C) blot. (D,E) Strip blots of immune-selected Type 1 (D) and Type 2 (E) vesicles probed with the indicated antibodies. (F) RNP in a 2.5 h, Cav1- and EEA1-depleted cytosolic fraction was selected with antibody against the indicated RIC subunit, treated with RNase in absence or presence of Triton X-100, as indicated. (G) RNP was immunoselected as in F, treated with Triton X-100 as indicated and analyzed by Western blot with anti-RIC1 or anti-RIC4A antibody. (H) Deduced positions of RIC subunits 1 (blue), 4A (pink) and 8B (yellow), and RNA (green) in the transport vesicle. Based on the model of RIC structure in Adhya (Adhya, 2008), and accessibility studies (see text).

Endocytic vesicles involved in intracellular transport are characterized by the presence of characteristic surface markers that may be used to identify and purify them. In view of the presence of RNP in the cytosolic fractions of transfected cells at intermediate times (Fig. 1), we isolated RNP-containing fractions from the cytosolic fraction by immunoprecipitation (IP) with non-immune or specific antibody. The efficiency and specificity of IP was checked by western blot analysis of the pellet and supernatant fractions of each immunoprecipitate. Cav1 was precipitated by anti-Cav1, but not by non-immune IgG, or by anti-Clathrin Heavy chain (Fig. 4A, upper); conversely, clathrin was pulled down by its cognate antibody only (Fig. 4A, lower). Little of no Cav1 was present in the supernatant after anti-Cav1 antibody pull-down, showing the IP to be quantitative for the endogenous Cav1 present in the fraction; similarly for Clathrin heavy chain (Fig. 4A). The efficiency and specificity of other antibodies was similarly confirmed (data not shown).

Cytosolic fractions from cells transfected with pcRNA1-R6 for 1 or 2 h were subjected to IP with anti-Cav1 or anti-RIC4A antibody; the supernatant from this first IP was subjected to a second IP with the other antibody; the pellets (P1 and P2) were subjected to western blot analysis. With non-immune IgG no signal was obtained, as expected (Fig. 4B). Anti-Cav1 antibody pulled down RIC4A from the 1 or 2 h fractions, indicating association of the carrier complex with Cav1. IP of the first supernatant from the 1 h fraction with anti-RIC4A failed to pull down any additional RIC4A, showing that all of the RNP is associated with Cav1 (Fig. 4B). However, the same sequence of antibody addition performed with the 2 h fraction revealed a significant amount of RIC4A signal after the second IP (∼30% of the total), indicating the presence of a Cav1^−^RNP^+^ species at this time (Fig. 4B). Northern blot analysis of these fractions similarly revealed the presence of pcRNA1 in Cav^+^ and Cav^−^ fractions at 2 and 3 h, with the Cav^−^ fraction amounting to ∼50% of the total (Fig. 4C). When the order of antibodies was reversed, i.e. anti-RIC4A followed by anti-Cav1, it was observed that there are two types of Cav^+^ compartments, one associated with RIC and the other not (Fig. 4B).

We purified cytosolic RNP after 1 h of transfection (Type 1 RNP) by immunoselection with anti-RIC4A antibody. Western blot analysis showed the presence of Cav1, FilA, Dyn2, NSF and the various RIC subunits, but the absence of clathrin or of the endosomal components EEA1, Rab5 and Rab7 (Fig. 4D). A second type (Type 2) of RNP was isolated from late (2–3 h) transfected cells by successive immuno-depletion of early and late endosomes, and of Cav1^+^ vesicles, before immunoselection with anti-RIC4A antibody. Type 2 RNP lacked Cav1, FilA and Dyn2 and had only traces of NSF (Fig. 4E).

The RNP recovered by immunoprecipitation from cytosolic fractions could be either free or in membrane-associated form. These states may be distinguished by testing the accessibility of the bound RNA to RNase, or of the individual RIC subunits to specific antibody. RNP in the cytosol of transfected cells was immunoselected with RIC subunit specific antibody, the immunoprecipitate was treated with RNase and assayed for cargo RNA by Northern blot. The RNA in the immunoprecipitate with anti-RIC4A or anti-RIC8B was resistant to RNase, but became sensitive on treatment with Triton X-100, indicating its entrapment in a membrane-bound compartment (Fig. 4F). No RNA was recovered with antibody against RIC1, to which the RNA binds (Fig. 4F), suggesting that this subunit, like the associated RNA, is inaccessible to the cytosol.

Western blot analysis of the immunoprecipitates confirmed this suspicion. RIC subunits were recovered with antibodies against RIC4A or RIC8B, but not those against RIC1 or RIC9 (Fig. 4G, upper). Treatment of the immunoprecipitates with Triton X-100 resulted in exposure of RIC1, with recovery of the complex by anti-RIC1 antibody (Fig. 4G, lower). Identical results were obtained with RNP from early (1 h, data not shown) or late (2.5 h) transfected cells (Fig. 4G). These observations imply that the RNP is asymmetrically orientated in membranous vesicles, with the RNA receptor (RIC1) and cargo located internally and membrane-associated subunits such as RIC4A and RIC8B exposed to the cytosol (Fig. 4H). The inaccessibility of specific RIC subunits or RNA to antibody or RNase respectively, and their exposure in presence of detergent, rules out the possibility that the RNP in either Type I or Type II vesicles is in the free, non-membrane associated form.

### Type 2 vesicles are sensitive to nocodazole but independent of EEA1

**Fig. 5.**
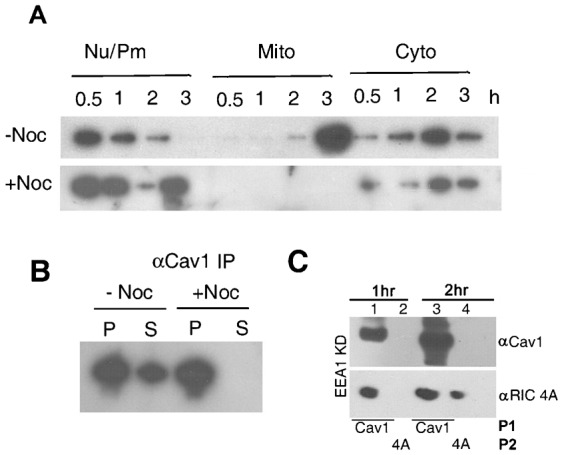
Effect of nocodazole (NOC) or EEA1 on subcellular distribution of pcRNA1 in HepG2 cells. (A) Indicated fractions from cells treated for indicated times with pcRNA1-RIC in the absence or presence of 10 µM NOC were Northern blotted and probed with anti-COII oligonucleotide. (B) The cytosolic fraction from cells treated for 2 h with pcRNA1-R8 in the absence or presence of NOC was immunoprecipitated with anti-Cav1 antibody and the pellets (P) and supernatant (S) were analyzed by Northern blot. (C) Sequential immunoprecipitates (anti-Cav1 followed by anti-RIC4A antibody) of 1- or 2-h cytosolic fraction from EEA1-KD cells were analyzed by Western blot.

The composition of Type 2 vesicles, lacking the components of caveolar endocytosis (Cav1, Dyn2 and FilA) suggests that they are derived from Type 1 vesicles, containing these components, by sorting. If so, formation of Type 2 vesicles should have similar or identical requirements to established sorting processes. Sorting of cargo from endosomes and their subsequent transport requires a functional microtubular network; thus, agents such as nocodazole that induce microtubule depolymerization inhibit the formation of SV40 tubular carriers (Pelkmans et al., 2001). In subcellular fractions from cells treated with pcRNA1 in presence of nocodazole, the RNA was bound to the plasma membrane and internalized to cytoplasmic compartments with about the same kinetics as in control cells. Between 2 and 3 h, however, there was a dramatic change in the intracellular location, with the RNA being associated with the plasma membrane/nuclear fraction instead of being targeted to mitochondria (Fig. 5A). Separation of the 2 h cytosolic fraction from normal or nocodazole-treated cells into Cav^+^ and Cav^−^ compartments by anti-Cav1 IP showed that whereas normally ∼40% of the vesicles were Type 2 after 2 h, nocodazole reduced Type 2 Cav^−^ vesicles to undetectable levels (Fig. 5B). Thus the formation of Type 2 vesicles, like that of viral sorted vesicles, is sensitive to nocodazole.

Sorting of viral cargo occurs in the EE or LE. It is possible that the Type 2 vesicles are derived by sorting in endosomes. To examine this possibility, the cytosolic fraction from normal or EEA1-KD cells transfected with pcRNA1-RIC for 1 or 2 h was subjected to sequential IP as above. In normal and EEA1-KD cytosolic fractions after 2 h, the proportions of Type 2 vesicles were comparable (27 vs. 32% of total) (Fig. 5C, compare with Fig. 4B), indicating that the lack of functional endosomes does not affect their formation.

### Transfer of RNP from Type 2 vesicles to mitochondria

**Fig. 6.**
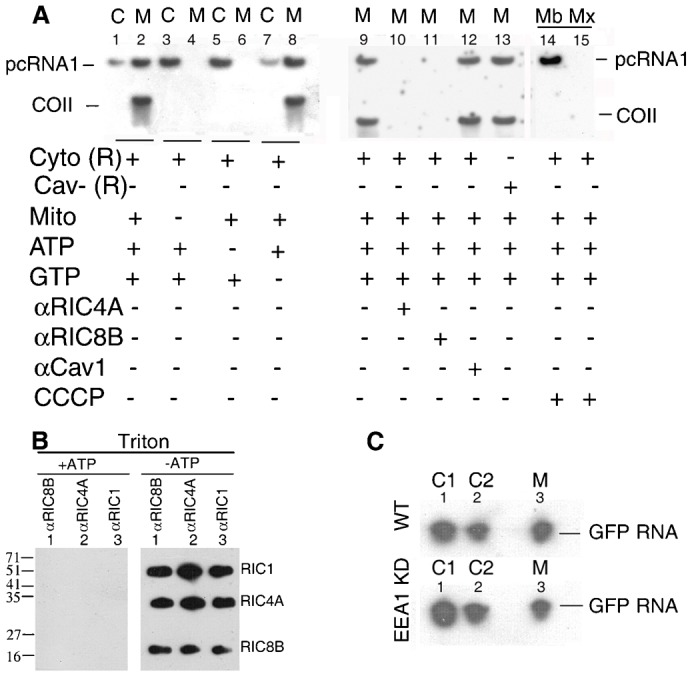
Transfer of RNP from cytosolic vesicles to mitochondria in vitro. (A) Transfer of pcRNA1 from post-caveosomal vesicles to mitochondria in vitro. The RNP-loaded cytosolic fraction (Cyto (R), 10 µl) from 2 h-pcRNA1-transfected FLP32.39 cells was incubated with mitochondria from untreated cells in the presence of ATP or GTP as indicated; Cav^−^ (R), Cyto (R) fraction immunodepleted of Cav1^+^ vesicles. After incubation, the reactions were centrifuged to separate the supernatant (C) and mitochondrial (M) fractions which were analyzed by Northern blot using anti-COII probe. In lanes 10–12, the Cyto (R) fraction was pre-incubated with the indicated antibody (1:200) before incubation with mitochondria. In lane 13, Cyto(R) was replaced by Cav^−^(R). Alternatively, mitochondria were pre-incubated with CCCP (50 µM, 10 min, 4°C) before incubation with Cyto(R) (lanes 14, 15). Re-isolated CCCP-treated mitochondria were subjected to freeze thaw lysis followed by centrifugal separation of the soluble matrix (Mx, lane 14) and insoluble membrane (Mb, lane 15) fractions. (B) The 2 h- cytosolic fraction from HepG2 cells transfected with RIC was incubated with mitochondria in the absence (lanes 1–3) or presence (lanes 3–6) of ATP, the mitochondria were re-isolated, solubilised with Triton X-100, and subjected to IP with the indicated antibodies. Immunoprecipitates were probed with a mixture of anti-RIC1, anti-RIC4A and anti-RIC8B. (C) The 2-h cytosolic fraction from normal (WT) or EEA1-KD cells transfected with GFP RNA was incubated with mitochondria under transfer conditions and the soluble (C2) and mitochondrial (M) compartments reisolated for Northern Blot.

While it is evident that Type 1 and Type 2 vesicles are present prior to the entry of RNP into mitochondria, it remained to be determined if such vesicles are true intermediates in the transport pathway. Therefore, we isolated the cytosolic fraction from HepG2 cells treated with pcRNA1-RIC complex for 2 h, and incubated it in presence of nucleotide co-factors with mitochondria from untreated FLP32.39 cells. The mitochondria were re-isolated by centrifugation and analyzed for the presence of RNA. Under these conditions the RNA was transferred from the cytosolic fraction to the mitochondria; this was evident from the appearance of COII mRNA by processing of pcRNA1 (Fig. 6A, lane 2). In control reactions lacking mitochondria, the RNA remained in the supernatant after centrifugation (Fig. 6A, lane 3), ruling out non-specific aggregation of the RNA-loaded vesicles during incubation. Transfer required ATP but not GTP (Fig. 6A, lanes 6, 8). Transfer was blocked by antibody against the subunits RIC4A or RIC8B, but not by anti Cav1 antibody (Fig. 6A, lanes 10–12). Since the 2 h post-mitochondrial fraction contains Type 1 as well as Type 2 vesicles (Fig. 4), we examined which of these was involved in the targeting process. Type 2 vesicles, isolated by immunodepletion of the Cav^+^ Type 1 vesicles, was as effective as the cytosolic fraction in the targeting assay, indicating these to be the true targeting vesicles (Fig. 6A, lane 13). In presence of the mitochondrial uncoupler CCCP, transfer to the mitochondrial membrane was not affected, but the pcRNA was bound to the mitochondrial membrane and not imported into the matrix, and hence not processed (Fig. 6A, lanes 14, 15). This is in keeping with the well known requirement of RIC-catalyzed RNA import for a transmembrane proton gradient (Bhattacharyya and Adhya, 2004).

To examine if some or all of the subunits of RIC are transferred during the reaction, transfer assays were performed with the 2 h cytosolic fraction from HepG2 cells, mitochondria were re-isolated, detergent-solubilized and immunoprecipitated with subunit-specific antibody. In presence of ATP, all of the RIC subunits were present in the material pulled down by any one of the antibodies (Fig. 6B, right); in the absence of ATP, none of the subunits was transferred (Fig. 6B, left). This indicates that the complex as a whole, rather than individual subunits, was transferred in vitro in an ATP-dependent process.

To determine whether the mitochondrial transfer function of the intermediate vesicles was affected in endosome-depleted cells, the transfer assay was performed with the cytosolic fraction from normal or EEA1-KD cells transfected with GFP RNA for 2 h (Fig. 6C). In reactions with the cytosolic fraction from normal cells, the transfer efficiency (i.e. the fraction of RNP transferred) was 83–86%; the corresponding value for cytosolic vesicles from EEA1-KD cells was 68%. Thus, lack of endosomes does not appreciably affect the transfer function of the vesicles.

### Dynamic sorting of Cav1 and RNP by fission and fusion

**Fig. 7.**
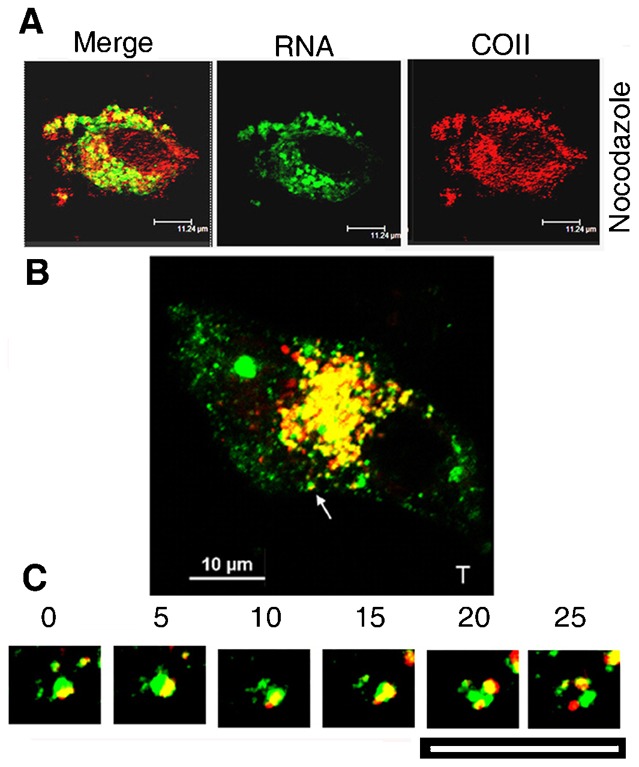
Sorting of RNP and caveolin in live cells. (A) A HepG2 cell transfected with AF488-labeled pcRNA1 (green) for 3 h in presence of 10 µM nocodazole and counterstained with anti-COII antibody (red). (B) Merged image of a HepG2 cell expressing Cav1-GFP (green) incubated with BODIPY-TR labeled pcRNA1-R8 complex (red) at 4°C then cultured for 2.5 h at 37°C in presence of 10 µM nocodazole. Cells were washed with drug-free medium and incubated for 30 min at 37°C before time-lapse imaging. The region around the single sorting vesicle (arrow) in each frame is enlarged in panel C. Note the splitting of a single vesicle to a Cav^−^ RNA-loaded transport vesicle (red), an empty Cav^+^ vesicle (green) and a smaller unsorted compartment (yellow). Scale bars: 12 µm (A); 10 µm (B,C).

To visualize the dynamics of the sorting process, BODIPY-TR labeled RNP was incubated with cells expressing Cav1-GFP, then treated with nocodazole to inhibit sorting (see above). Treatment with nocodazole resulted in the predominance of large RNP vesicles (Fig. 7A). Following removal of the inhibitor to re-form the microtubules and allow the synchronized initiation of sorting, numerous Cav^+^RNA^+^ vesicles with tubular Cav^−^RNA^+^ protrusions were concentrated at the nuclear periphery (Fig. 7B). The RNP vesicles exhibited considerable structural alterations, forming transient multi-lobed structures and protruding and retracting extensions. Separation of the RNP into sectors and channels inside the vesicle was apparent, with occasional formation of Cav1-free RNP buds. Spherical RNP and empty Cav1 vesicles were released by dissociation of larger vesicles (Fig. 7C; supplementary material Movie 1), and occasionally fused back with them. There was no evidence of tubular vesicles. Thus the RNP vesicles are dynamic structures undergoing internal reorganization, fission and fusion. In the time sequence shown, RNA was restricted to a sector of a Cav1^+^ RNP vesicle; a RNA^+^Cav^−^ protrusion appeared at one point of the surface of the ovoid vesicle and progressively elongated; simultaneously, RNA was observed to concentrate in the diametrically opposite region; finally the vesicle split into 3 particles: a RNA-bearing vesicle, an empty Cav^+^ vesicle, and a RNA-loaded Cav^−^ vesicle (Fig. 7C). The estimated sorting time in this case was ∼20 s.

## DISCUSSION

In this report we present evidence that RIC is taken up by a Cav1-dependent mechanism and transported to mitochondria by a multi-step process that appears to be distinct from classical pathways through the early or late endosome.

Employing a synchronized protocol, a high multiplicity of transfection (several thousand RNP molecules per cell), and subcellular fractionation, we were able to show that trafficking of RNP consists of a number of discrete steps including formation of Cav1^+^ vesicles from the plasma membrane, formation of Cav^−^ vesicles, and vesicle-to-mitochondrion transfer. By sequential immunoprecipitation we were able to detect different types of RNP vesicles in the cytosolic fraction (Fig. 4). The Type 2 particles correspond to the Cav^−^RNA^+^ vesicles observed by microscopy (Fig. 3); they lack Cav1 and associated factors (Fig. 4), and contain asymmetrically orientated RIC subunits and entrapped RNA (Fig. 4); their formation is dependent on the microtubular network (Fig. 5); and they are capable of transferring their cargo to mitochondria (Fig. 6). A reasonable conclusion is that Type 2 particles represent sorted vesicles directly targeted to mitochondria.

We describe here an in vitro assay for vesicle-to-mitochondrion transfer of RNP, using purified cargo-loaded vesicles. Remarkably, transfer was dependent on ATP but not GTP (Fig. 6). This is unusual, since fusion of vesicles requires GTP hydrolysis by the Rab GTPases (Zerial and McBride, 2001), and suggests a distinct mode of transfer of RNP to the mitochondrial surface, e.g. escape from the vesicle followed by re-insertion, as is thought to occur for certain viruses (Farr et al., 2005).

In contrast to the variety of uptake mechanisms described, there appears to be a general consensus for the presence of only a single sorting compartment: the endosome (EE or LE). The endosomal transport pathway, involving Rab5 and Rab7, is by far the one most commonly used by eukaryotic viruses, as shown by a recent survey (Mercer et al., 2010). Work presented here supports the existence of a non-endosomal sorting compartment in mammalian cells. (1) Transport of RNP to mitochondria was not affected by knockdown of essential endosomal components Rab5, EEA1 and Rab7. (2) RNP vesicles that are negative for endosomal markers were formed during trafficking. (3) Transferrin-laden endosomes were negative for RNP. (4) Formation of Type 2 vesicles was not affected by depletion of endosomes. (5) The Type 2 vesicles formed in endosome-depleted cells were capable of transferring cargo to mitochondria. (6) The mechanics of sorting appeared to be different from those described for EE and LE. An alternative pre-existing organelle, the caveosome, was originally envisaged in studies of caveolin-dependent endocytosis of SV40 virus (Pelkmans et al., 2001), but subsequently denied, being equated with late endosomes (Parton and Howes, 2010; Engel et al., 2011). We propose that caveolar cargo such as RIC-RNP and cholera toxin may involve a transient, rather than pre-existing, non-endosomal sorting compartment.

Productive transport of Adenovirus Type 2/5 (Gastaldelli et al., 2008) or of arenaviruses (Quirin et al., 2008; Rojek et al., 2008) is independent of Rab5, though there is some co-localization of the virus with endosomes (Gastaldelli et al., 2008). Similarly, we show here that, although the majority of RNP was targeted to mitochondria in a Rab5-independent manner, a minority of RNP endocytic vesicles were shunted to endosomes and degraded in ILVs (Fig. 3); however, endosomal uptake of RNP was absent in Rab5-deficient cells (Fig. 3), while transport to mitochondria was normal (Fig. 2), and no free (i.e. non-membrane associated) RNA or RIC was detectable in the cyto (Fig. 4), arguing against the possibility of endosomal entry of RNP and escape by a Rab5-independent route, as suggested for adenovirus (Gastaldelli et al., 2008). Thus, productive and non-productive transport pathways may compete for the same endocytic vesicles, accounting for the presence of Ad2/5 or other cargo in endosomes.

Live cell observations revealed that Cav1^+^ vesicles undergo dynamic intravesicular rearrangements leading to formation of RNP-enriched sectors, followed by fission to release the coat protein (Cav1) and RNP vesicles (Fig. 7; supplementary material Movie 1). Large Cav1^+^EEA1^−^ vesicles have been observed during trafficking of CTX-B, and the sorting observed by videography involved budding of spherical vesicles from such bodies (Nichols, 2002), resembling the sorting of RNP we report here. Two types of endosomal sorting have so far been described. The first involves the formation of ILVs within EE in a process regulated by the ESCRT complex (Rusten et al., 2012). The second envisages extrusion of tubular vesicles form LE in a Retromer-mediated process (Cullen and Korswagen, 2012). Sorting of RNP appears to be distinct from either type: sorting occurred in endosome-depleted cells (Fig. 5), spherical rather than tubular vesicles were formed, and the sorting vesicles observed live cell imaging do not resemble ILVs (Fig. 7; supplementary material Movie 1), although Cav^+^ ILVs devoid of RNA are formed at late times (Fig. 3B,C), and probably represent empty caveolar vesicles in the process of recycling. We also observed reverse sorting, i.e. fusion of RNP vesicles back to LRV. Reversible interactions in time and space (i.e. within the same vesicle) imply that at any moment the frequency of sorting is the net result of fusion and dissociation of the transport vesicles, as opposed to the ordered progression proposed for endosomal sorting events. The molecular details of this novel sorting process remain to be worked out.

## MATERIALS AND METHODS

### Preparation of RNA

Polycistronic RNA1 (pcRNA1, 4.1 kb) contains the contiguous stretch of human mitochondrial DNA between the COI and COIII genes, and includes a 5′-terminal tag for attachment to the carrier complex (Mahato et al., 2011). The full-length GFP gene from pZS Green (Clontech Laboratories, Mountain View CA, USA) was amplified using coding region sense and antisense primers and ligated to a T7 promoter-tag cassette before re-amplification with terminal primers, as described (Mahato et al., 2011). pcRNA1 or tagged GFP RNA was prepared by in vitro transcription of the appropriate DNA template. For fluorescence microscopy, RNA was labeled with Alexa Fluor (AF) 488, AF546 or BODIPY-Texas Red (TR) using the appropriate fluorophore-conjugated nucleoside triphosphate precursor (Mahato et al., 2011).

### Preparation of carrier complex

R6 and R8 are functional sub-complexes of RIC containing 6 or 8 nucleus-encoded subunits from *L. tropica*; these were obtained by reconstitution from purified subunits expressed in *E. coli* (Mukherjee et al., 2007). Carrier complex was combined with tagged RNA to form RIC-RNP (Mahata et al., 2006).

### Cell lines

The hepatocarcinoma line HepG2 containing wild-type mitochondrial (mt) DNA was cultivated as monolayers in DMEM medium with 10% Fetal Bovine Serum (FBS). The cybrid line FLP32.39 is homoplasmic for a 1.9 kb mtDNA deletion covering the COII–COIII region (Sobreira et al., 1999) resulting in lack of the corresponding transcripts and a general down regulation of mitochondrial translation (Mahata et al., 2006).

### Transfection protocol

Before administration, the RNA-protein complex (RNP) was diluted into sterile phosphate buffered saline (PBS), or equivalent buffer, or culture medium, to a concentration of 1 pmol of RNA/16 µg R6 or R8 protein/ml. Semi-confluent cell monolayers were incubated with pcRNA1:R8 complex for 30 min at 4°C, the medium was replaced with fresh medium lacking RNP, and the cells were incubated for various times at 37°C under 5% CO_2_. At the indicated times, cells were washed with PBS and harvested for subcellular fractionation, or the medium was replaced with PBS containing 4% paraformaldehyde for fixation.

### Reporter expression

Cells were transfected with plasmid constructs (50 µg) encoding Cav1-GFP (pCav1-mEGFP) (Hayer et al., 2010) or α-tubulin-GFP (pAcGFP1-tubulin; Clontech Laboratories, Mountain View CA, USA) using Lipofectamine 2000 (Sigma–Aldrich Corp., St. Louis, MO, USA), and incubated for 48 h before RNP treatment. To mark mitochondria, cells were infected with a baculovirus encoding Red Fluorescent Protein (RFP) tagged with a mitochondrial targeting sequence (Organelle Lights Mito-RFP, Life Technologies, Grand Island, NY, USA) for 24 h. Transfections or infections were performed as per the respective manufacturer's instructions.

### RNA interference

For the biochemical fractionation experiment, double-stranded small interfering (si) RNAs targeting Cav1 or clathrin heavy chain, or single-stranded caveolin-1 plus strand (control) were prepared as described (Mahata et al., 2006) (supplementary material Table S1). FLP32.39 cells (2–2.5×10^6^) were transfected with dsRNA (50 pmol) complexed with Lipofectamine 2000 (50 µg) for 22 h, then washed and incubated with pcRNA-R8 complex for 3 h before subcellular fractionation. For live cell imaging, siRNAs against known targets were designed and supplied by Sigma–Aldrich Corp., St. Louis, MO, USA. Semi-confluent HepG2 cells were transfected with siRNA (20 pmol per chamber) using Lipofectamine 2000, and cultured for 72 h before treatment with RNP. Untreated or siRNA-treated FLP32.39 cells were transfected with pcRNA1 and the O_2_ uptake rate measured after 6 h, as described (Mahato et al., 2011). HepG2 cells were transfected with GFP RNA and imaged after 6 h.

### Inhibitors

HepG2 cells were transfected with Mito-RFP baculovirus as above. Transfected cells were incubated with nystatin (25 µg/ml) plus progesterone (10 µg/ml), or chlorpromazine (14 µM) for 1 h at 37°C, then AF488-RNP (1 pmol) was added and incubation continued for 6 h before microscopic observation. Alternatively, cells were treated with 10 µM nocodazole in culture medium for 1 h at 37°C, before addition of RNP and incubation for 2.5 h. The cells were then washed with medium lacking nocodazole or RNP, and incubated for a further 30 min (to allow recovery from nocodazole-induced disruption of microtubular structure) before observation.

### Co-transport assays

Fluorescent-tagged cholera toxin B subunit (AF647-CTXB), bovine serum albumin (AF647-BSA) and transferrin (AF633-Tfn) were obtained from Molecular Probes (Life Technologies, Grand Island, NY, USA). HepG2 cells were incubated with a mixture of AF488-RNP (1 pmol/ml) and AF647-BSA or AF633-Tfn (10 µg/ml) for 1 h at 4°C. Alternatively, cells were incubated with AF488-RNP for 45 min at 4°C before addition of CTXB and continuation for another 15 min. Cells were then transferred to 37°C for the indicated time before observation.

### Confocal microscopy

For live cell imaging, cells growing in Lab Tek chambers were placed in the on-stage incubator (Tokai Hit, Shizuoka-ken, Japan) of the microscope set at 37°C, 5% CO_2_ for direct observation. Fixed cells were incubated with the indicated primary antibody (supplementary material Table S2) and AF-labelled secondary antibodies (supplementary material Table S2). Cells were imaged with a Nikon (Tokyo, Japan) A1R confocal imaging system using 488 and 638-nm lasers and NIS Elements AR 3.0 software. Co-localization was expressed as Pearson's Correlation Coefficient (PCC), and per cent uptake was measured as the fraction of total cell-associated fluorescence in the cytoplasm. Time-lapse imaging was performed at 4–5 s intervals on a single focal plane at 512×512 resolution. GFP fluorescence (average intensity/cell; n = 35) was measured as Regions of Interest (ROI) using the same software.

### Preparation of subcellular fractions

Plasma membrane/nuclear, mitochondrial and post-mitochondrial fractions were obtained by differential centrifugation, as described (Mahato et al., 2011). Homogenates of normal or RNA-treated cells were separated into plasma membrane/nuclear, mitochondrial and post-mitochondrial fractions by differential centrifugation. Normal or RNA-treated cells (2–2.5×10^6^) were harvested, washed and incubated in 3× packed cell volume (150 µl) of Lysis Buffer (3.5 mM Tris-HCl, pH 7.8, 2 mM NaCl, 0.5 mM MgAc_2_) for 15 min at 4°C, homogenized in an all-glass homogenizer with 10–15 strokes, checked for cell lysis, then 1/9th volume (∼12 µl) of 350 mM Tris-HCl, pH 7.8, 0.2 M NaCl, 50 mM MgAc_2_ was added and the homogenate was centrifuged at 4200 rpm for 5 min at 4°C in a microcentrifuge (Hereaus model Fresco). The pellet, containing nuclei and plasma membrane vesicles, was separated from the supernatant by aspiration, and the latter was spun at 13,000 rpm for 5 min to separate the pellet of mitochondria (M) from the post-mitochondrial or cytosolic fraction (C). Western blot analysis with marker antibodies was used to confirm fraction identities. Ribonuclease (RNase) treatment of subcelluar fractions (10 µl) was carried out in 20 µl of 10 mM Tris-HCl, pH 7.4, 5 mM MgAc_2_, 1 mM DTT, 0.32 M sucrose, 2.5 µg/ml RNase A, 50 units/ml RNase T1 for 15 min at 37°C. Then 0.4 ml of 4M guanidinum isothiocyanate, 25 mM sodium citrate, 0.5% sarkosyl, 0.1% β-mercaptoethanol, 4M lithium chloride was added and processed for RNA preparation.

### Immunoprecipitation

The appropriate subcellular fraction (10 µl) was incubated with 20 µl of blocking buffer (PBS–1% bovine serum albumin) and 5 µl of 1:50 dilution of specific antibody (final 1:350; supplementary material Table S2) in blocking buffer, for 1 h at 4°C. Then 20 µl of Protein A Sepharose beads (GE Healthcare, Pittsburgh, PA, USA, 1:1 suspension in blocking buffer) was added and incubation continued for 1 h. The beads were separated by low-speed centrifugation (500 rpm, 5 min). Type 1 vesicles were selected with anti-RIC4A antibody from cytosolic fraction of HepG2 cells transfected with pcRNA1-R6 for 1 h; Type 2 vesicles were obtained by selection with anti-RIC4A antibody from the 2 h-cytosolic fraction that had been successively immunodepleted of EEA1, LAMP1 and Cav1.

### Vesicle-to-mitochondrion transport assay

Post-mitochondrial supernatant (Cyto(R) was prepared as above from ∼2×10^6^ HepG2 cells treated for 2 h with 1 pmol of pcRNA1-R8 complex, while mitochondrial fraction (M) was prepared from a similar number of untreated cells. Cyto (R) was immunodepleted of Cav^+^ vesicles using anti-Cav1 antibody to yield the Cav^−^ (R) supernatant. Complete transport reactions (30 µl) contained 10 mM Tris-HCl, pH 7.5, 10 mM MgAc_2_, 2 mM DTT, 1 mM ATP, 1 mM GTP, 10 µl of Cyto(R) and 10 µl of M. After 1 h incubation at 37°C, the reaction mixture was centrifuged at 13,000 rpm for 5 min to re-pellet the mitochondria. RNA was prepared from the washed pellet and supernatant for analysis.

### Protein analysis

Western blots were probed with appropriate antibodies (supplementary material Table S2) using the ECL system (GE Healthcare, Pittsburgh, PA, USA).

### Preparation and analysis of cellular RNA

Northern blots on Hybond N+ (GE Healthcare, Pittsburgh, PA, USA) were probed with ^32^P-labeled primer complementary to the COII or GFP gene (supplementary material Table S3). Band intensities were quantified by densitometry of autoradiograms.

### O_2_ measurements

O_2_ uptake by stirred cell suspensions was monitored using a Clarke-type electrode (Mahato et al., 2011).

## Supplementary Material

Supplementary Material
