# Supplementary material for: Vesicular transport of a ribonucleoprotein to mitochondria
Source: Biol Open. 2014 Oct 17;3(11):1083–91. doi: 10.1242/bio.20149076 (PMC4232766; doi:10.1242/bio.20149076)
Supplement: Supplementary Material [file supp_3_11_1083__index.html]

Vesicular transport of a ribonucleoprotein to mitochondria — Supplementary Material 

# Vesicular transport of a ribonucleoprotein to mitochondria

## bio.20149076 Supplementary Material

**Files in this Data Supplement:**

- Supplementary Material - Joyita Mukherjee et al. doi: 10.1242/bio.20149076
- Movie 1 - **Movie 1. A HepG2 cell expressing Cav1-GFP (green) incubated with BODIPY-TR labeled pcRNA1-R8 complex (red) at 4°C then cultured for 2.5 h at 37°C in presence of nocodazole.** Cells were washed with drug-free medium and incubated for 30 min at 37°C before imaging.
