# Supplementary material for: Vesicular transport of a ribonucleoprotein to mitochondria
Source: Biol Open. 2014 Oct 17;3(11):1083–91. doi: 10.1242/bio.20149076 (PMC4232766; doi:10.1242/bio.20149076)
Supplement: Supplementary Material [file supp_bio.20149076_bio.20149076-s1.pdf]

Supplementary Material
Joyita Mukherjee et al. doi: 10.1242/bio.20149076

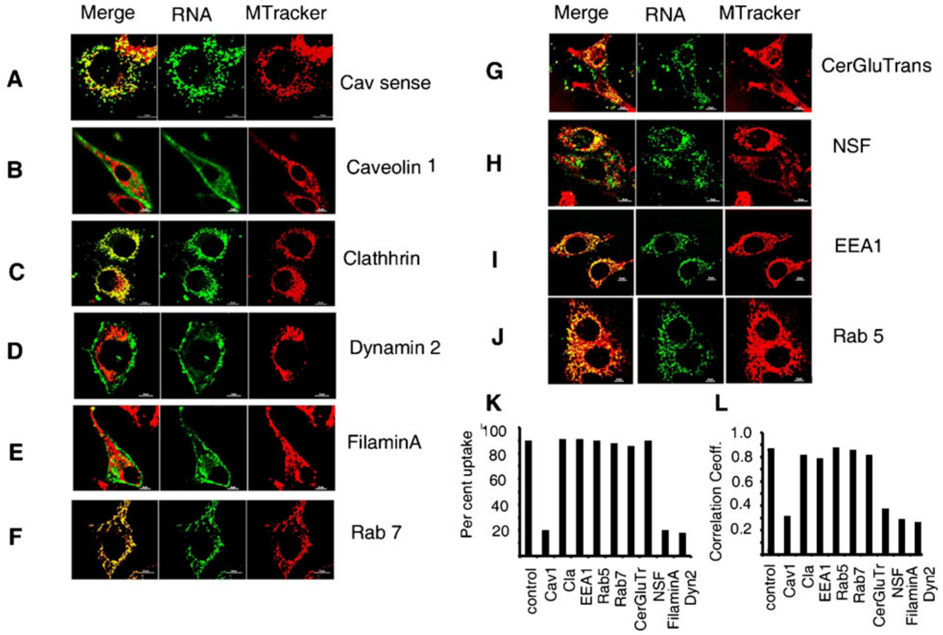

Fig. S1. Effect of RNA interference on uptake and mitochondrial targeting of RNP. (A–J) HepG2 cells pre-treated with Cav1 sense or siRNA against the indicated target Cav1 were incubated for 6 h with AF488-labeled pcRNA1-R8 complex (green), counterstained with MitoTracker Deep Red and imaged live. Co-localization is indicated as yellow areas in the merged image. (K) Per cent uptake of AF488-RNA in cells treated with the indicated siRNAs. (L) Pearson's Correlation Coefficient for the two fluorophores in siRNA-treated cells. Scale bars: 10 µm.

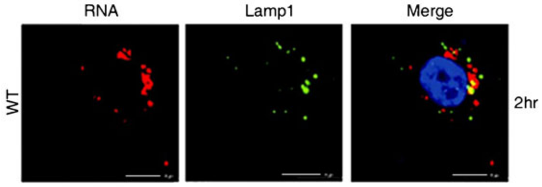

Fig. S2. Non-localization of RNP with late endosomes. HepG2 cells were transfected with AF546-labeled pcRNA1 (red) for 2 h. Fixed cells were stained for LAMP1 (green).

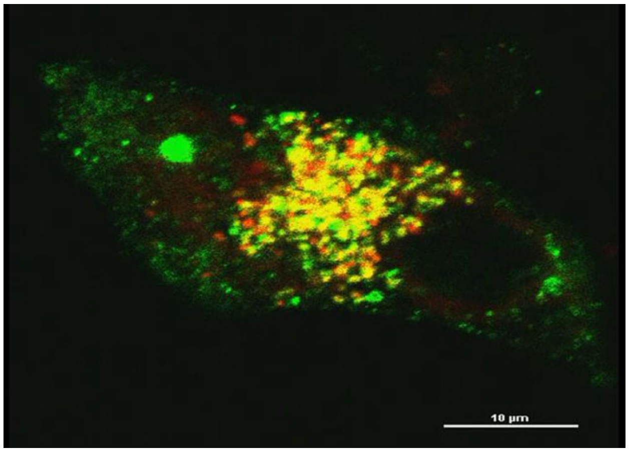

Movie 1. A HepG2 cell expressing Cav1-GFP (green) incubated with BODIPY-TR labeled pcRNA1-R8 complex (red) at 4˚C then cultured for 2.5 h at 37˚C in presence of nocodazole. Cells were washed with drug-free medium and incubated for 30 min at 37˚C before imaging.

Table S1. SiRNA list

Si-RNA NO.	NAME OF Si-RNA	SEQUENCE (5′–3′)
1.	Caveolin-1	AACCAGAAGGGACACACAGUU
2.	Clathrin HC	UAAUCCAAUUCGAAGACCAAU
3.	Caveolin(sense)	AACUGUGUGUCCCUUCUGGUU
4.	Dynamin-2	CCCUUGACACCAUCCUGAA
5.	Early Endosomal Antigen-1(EEA1)	GCAUCUAAAGGCGGAGUUU
6.	Filamin A	CAGUCAAGUUCAACGAGGA
7.	Rab5A	GUCCUAUGCAGAUGACAAU
8.	Rab7	GUGUUAUUAUGUUUGACGU
9.	CerGluTransferase	GCUCAUAUUCAAUUUCUCA
10.	NSF	CCAAGAAGAUUAUGCAAGU

Biology Open

Biology Open (2014) 000, 1–9 doi:10.1242/bio.20149076

Table S2. Antibodies

ANTIGEN	SOURCE	TYPE	METHOD	DILUTION
CAVEOLIN 1	BD bioscience	Mouse IgG2a	IP	1:50
			WB	1:1000
			IF	1:350
CLATHRIN HEAVY CHAIN	BD bioscience	Mouse IgG1	IP	1:50
			WB	1:1000
RIC 1	G.E lab	Mouse polyclonal	IP	1:50
			WB	1:1000
RIC9	G.E lab	Mouse polyclonal	IP	1:50
			WB	1:1000
RIC4A	G.E lab	Mouse polyclonal	IP	1:50
			WB	1:1000
RIC8B	G.E lab	Mouse polyclonal	IP	1:50
			WB	1:1000
RIC 8A	G.E lab	Mouse polyclonal	IP	1:50
			WB	1:1000
COII	BD bioscience	Mouse IgG1	IF	1:350
Early Endosomal Antigen-1(EEA1)	BD bioscience	Mouse IgG1	IF	1:350
			WB	1:1000
PCNA(F-2)	Santa Cruz Biotechnology	Mouse monoclonal antibody	WB	1:1000
			IF	1:350
Na/K ATPase(Alpha subunit)	Sigma	Mouse monoclonal IgG2b	WB	1:1000
GAPDH	Abcam	Rabbit Polyclonal IgG	WB	1:1000
F1 ATP synthese subunit β	GE lab	Mouse polyclonal	WB	1:1000
Actin	Millipore	Rabbit Polyclonal IgG	WB	1:1000
Filamin A	Abcam	Rabbit polyclonal IgG	WB	1:1000
Rab5	Abcam	Rabbit polyclonal IgG	WB	1:1000
			IF	1:350
Rab 7	Abcam	Mouse monoclonal IgG2b	WB	1:1000
NSF	Invitrogen	Rabbit polyclonal IgG	WB	1:1000
Dyn2	Abcam	Rabbit polyclonal IgG	WB	1:1000
CerGluTransferase	Abcam	Mouse IgG	WB	1:1000
Lamp1	BD bioscience	Anti mouse IgG2b	WB	1:1000
			IF	1:350
AF 633 secondary antibody	BD bioscience	Anti mouse/anti rabbit IgG	IF	1:500
AF 488 secondary antibody	BD bioscience	Anti mouse/anti rabbit IgG	IF	1:500
Secondary antibody AP Conjugated	Sigma	Anti mouse IgG/anti rabbit IgG	WB	1:4000

Table S3. Oligoneucleotides

OLIGO NO.	NAME OF GENE	SEQUENCE (5′–3′)
1	pcRNA1	ACCTACTTGCGCTGCATGTGCCAT
2	GFP	CGGTCGACCTAGGGCAATGCAGATCC
3	COII	ACCTACTTGCGCTGCATGTGCCAT
